# Novel cooling vest with personal protective equipment alleviates heat strain without increasing metabolic demands in the heat

**DOI:** 10.5271/sjweh.4261

**Published:** 2026-01-01

**Authors:** Patarawadee Sainiyom, Vitoon Saeangsirisuwan, Clarence Hong Wei Leow, Jason Kai Wei Lee, Juthamard Surapongchai

**Affiliations:** 1 Faculty of Physical Therapy, Mahidol University, Nakhon Pathom, Thailand.; 2 Department of Physiology, Faculty of Science, Mahidol University, Bangkok, Thailand.; 3 Human Potential Translational Research Programme, Yong Loo Lin School of Medicine, National University of Singapore, Singapore.; 4 Department of Physiology, Yong Loo Lin School of Medicine, National University of Singapore.; 5 Heat Resilience and Performance Centre, Yong Loo Lin School of Medicine, National University of Singapore, Singapore.

**Keywords:** carbon composite, climate change, cooling modality, heat stress, metabolism, PPE

## Abstract

**Objective:**

Wearing medical personal protective equipment (PPE) substantially increases heat strain by elevating metabolic heat production while impairing heat dissipation. Cooling vests are a practical countermeasure, yet their efficiency depends on thermal conductivity and comfort. This study examined the thermoregulatory and perceptual responses to PPE use and evaluated the efficacy of a novel carbon-based cooling vest with enhanced heat transfer capacity.

**Methods:**

A randomized crossover design was employed in which 12 participants completed 100 minutes of simulated healthcare activity in a climatic chamber (32 °C, 70% RH) under three conditions: medical scrubs (NoPPE), scrubs with PPE (PPE), and scrubs with PPE plus the cooling vest (PPE+Vest). Physiological, thermoregulatory, and perceptual variables were continuously monitored across conditions.

**Results:**

Compared with PPE alone, PPE+Vest markedly attenuated heat strain, lowering core temperature [PPE 38.4, standard deviation (SD) 0.4, ^0^C versus PPE+Vest 37.5 (SD 0.4) ^0^C, P=0.001] and heart rate [PPE 123 (SD 11) bpm versus PPE+Vest 107 (SD 15) bpm, P<0.001], while improving thermal sensation [PPE 2.0 (SD 0.8) versus PPE+Vest 0.8 (SD 0.8), P=0.006]. These thermoregulatory benefits occurred without an increase in metabolic energy expenditure [PPE 317 (SD 50) kcal versus PPE+Vest 317 (SD 53) kcal, P=0.891].

**Conclusions:**

The novel carbon-based cooling vest effectively suppressed heat storage by enhancing conductive heat transfer, leading to core and skin temperatures comparable to NoPPE. Importantly, despite its additional weight, the vest did not impose extra metabolic demands, offering a practical strategy to maintain thermal comfort and physiological stability during prolonged medical work in hot environments.

Healthcare workers may wear full-body personal protective equipment (PPE) for extended periods in specific high-risk situations, such as when treating patients with airborne or highly contagious infections in isolation wards, field hospitals, or during a decontamination procedure. While PPE provides essential protection, it limits evaporative and convective heat loss (1). In hot and humid environments, this can result in progressive heat accumulation, leading to thermal strain. Elevated body core temperature (T_c_), discomfort, reduced concentration, fatigue, and impaired task performance were associated with a decline in medical and patient safety (2, 3). These outcomes are particularly relevant for healthcare personnel who perform critical tasks under time pressure, where heat strain could compromise concentration, efficiency, and well-being (2, 4).

To counteract such risks, cooling vests represent a practical solution that does not interfere with workers’ external environment. The benefits of cooling vests help sustain work performance, reduce discomfort, and enhance tolerance during prolonged PPE wearing in hot environments (5, 6). Previous approaches using ice-based, phase change material (PCM) and liquid cooling vests have demonstrated effectiveness in alleviating heat strain by reducing T_c_, skin temperature (T_sk_), heart rate (HR), and heat storage (7–9). However, their duration of cooling and practicality may be limited (5, 8). In addition, their effectiveness remains inconsistent, with some studies showing significant T_c_ reductions while others report minimal or no improvement (10–12). These discrepancies may stem from differences in study protocols, individual metabolic responses, or the cooling vest coolant’s mechanism and designs.

Traditional ice- or water-based as PCM cooling vests operate primarily through sensible heat transfer, by absorbing heat as the ice melts or warms. While ice contains a high latent heat, enabling it to absorb significant amounts of heat, its low thermal conductivity and very low temperature can induce discomfort and potential tissue irritation upon prolonged skin contact (12, 13). In contrast, PCM cooling vests utilize materials that absorb and release heat during phase transitions, providing a more sustained and controlled cooling effect (13, 14). Other PCM, such as paraffin hexa- and tetra-decane, have moderate latent heat and melting points closer to skin comfort ranges (7, 15). However, their low thermal conductivity can slow heat absorption, leading to delayed activation, limited cooling duration, and uneven cooling (7, 13). To enhance the performance of PCM cooling vests, a novel carbon-based additive has been developed. Carbon materials, such as expanded graphite and graphene, have been incorporated into PCM composites to improve thermal conductivity (16, 17). These additives form a conductive network within the PCM, facilitating faster and more uniform heat transfer, thereby enhancing the cooling efficiency and prolonging the cooling duration (18). The integration of carbon-enhanced PCM into cooling vests offers a promising solution for environments where mobility and practicality are critical (19). However, the physiological and metabolic impacts of wearing such enhanced cooling vests remain under investigation. Further research is essential to understand the cooling benefits associated with wearing a novel carbon-based PCM cooling vest with PPE in hot environments.

Therefore, the present study aimed to evaluate the physiological and metabolic effects of wearing a carbon-based cooling vest with PPE under hot conditions. By comparing responses across conditions with and without PPE and cooling, we hypothesized that this novel vest could effectively reduce heat accumulation and maintain thermal balance in healthcare-relevant settings.

## Methods

### Participants

Six male and six female participants were recruited in this study [mean age 20.8, standard deviation (SD) 1.2] years [males: 20.2 (SD 1.0) years, females: 21.3 (SD 1.0) years], body mass: 60.3 (SD 6.5) kg [males: 64.3 (SD 6.0) kg, females: 56.4 (SD 4.4) kg, P=0.028], height: 165 (SD 7) cm [males: 171 (SD 4) cm, females: 159 (SD 4) cm, P=0.001], body mass index: 22.1 (SD 1.7) kg/m^2^ [males: 21.9 (SD 1.2) kg/m^2^, females: 22.4 (SD 1.2) kg/m^2^], mean body fat percentage: 23 (SD 9) % [males: 15 (SD 2) %, females: 30 (SD 1) %, P<0.001], lean body mass (LBM): 44.1 (SD 7.6) kg [males: 51.1 (SD 2.0) kg, females: 37.1 (SD 2.0) kg, P<0.001], body surface area (BSA): 1.7 (SD 0.1) m^2^ [males: 1.8 (SD 0.1) m^2^, females: 1.6 (SD 0.1) m^2^, P=0.002], and BSA to LBM ratio: 3.8 (SD 0.4) m^2^/kg·10^2^ [males: 3.4 (SD 0.1) m^2^/kg·10^2^, females: 4.2 (SD 0.1) m^2^/kg·10^2^, P<0.001]). All participants were healthy and reported to have a sedentary lifestyle or participated in light-intensity exercise for ≤30 minutes and ≤3 times per week (20). The Centre for Ethical Reinforcement of Human Research at Mahidol University approved all research protocols (MU-CIRB 2023/377.1812, COA 2024/009.1701).

### Experimental protocol

**Figure 1:**
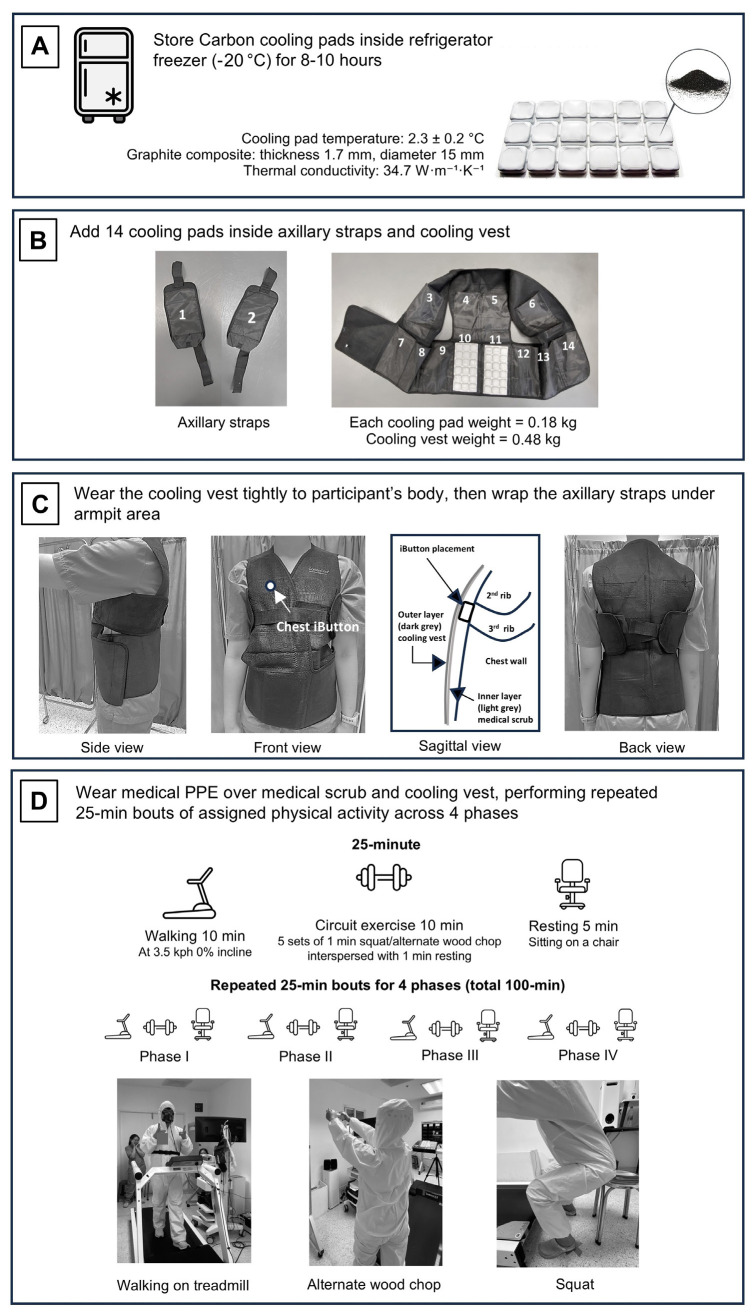
Study procedure. A. the preparation and specification of cooling pads, B. the placement of 14 pads into axillary straps and a cooling vest, C. how participants wear the cooling vest and straps, including the correct placement of an iButton sensor, and D. the 100-minute physical activity protocol, which consists of four 25-minute bouts of walking, circuit exercises, and rest while wearing medical PPE.

All participants completed three experimentals in a randomized and counterbalanced manner while wearing three different attires: (i) twill weave fabric medical scrub (NoPPE), (ii) medical scrub with PPE (3M™ Disposable Protective Coverall 4510; 3M, Berkshire, UK) (PPE), and (iii) medical scrub with PPE underneath a cooling vest (Comfort Suit V™; Global Healthcare, Singapore) integrated with cooling pads (Emcools Flex.Pad™, Global Healthcare, Singapore) (PPE+Vest). The cooling vest consisted of 14 cooling pads (each weighing 0.18 kg) with the vest (weighing 0.48 kg). The total weight of the cooling vest was 3.0 kg. The preparation and wearing processes of the cooling vest are illustrated in figure 1A–1C. The total weight of the attire was 0.94, 1.13, and 4.13 kg for NoPPE, PPE, and PPE+Vest, respectively. Each trial was conducted at the same time of day to control circadian effects and was separated by at least one week to ensure full recovery. To minimize potential menstrual cycle effects on study validity, only female participants who were not in their menstrual phase were included in the experimental sessions. The data was collected in an environmental chamber set at a dry bulb temperature (T_db_) and relative humidity (RH) of 32°C and 70%, representing a tropical climate setting (21).

Participants were instructed to maintain a consistent sleep schedule and diet throughout the study, and to avoid strenuous exercise for 24 hours before each experimental trial. Participants ingested a temperature capsule (e-Celsius Performance; BodyCap, Herouville Saint-Clair, France) 6–8 hours before each trial. On the day of the trial, a mid-stream urine sample was collected upon arrival, and hydration status was assessed using a hand-held refractometer (PAL-10S; ATAGO®, Saitama, Japan). A urine-specific gravity of <1.020 indicated euhydration and was required before each trial could begin. During each trial, participants engaged in 100 minutes of medical work-simulated physical activity [1.5–4.0 metabolic equivalents (MET)] (22), which consisted of four 25-minute phases (phases I–IV). Each 25-minute phase consisted of 10 minutes of walking, followed by a 10-minute circuit exercise mimicking large muscles used during patient care and a 5-minute rest, as shown in figure 1D. During the circuit exercise, participants engaged in 1-minute resistance workouts (squats or alternate wood chops to right or left sides) holding 1.5 kg dumbbell, followed by a 1-minute rest for five consecutive rounds. The rhythm of the circuit exercise was controlled using a metronome at 30 beats per minute. Visual cues, such as markers on the floor and wall, were used to regulate arm and knee movement during squats and alternate wood chops. At the end of the trial, participants in PPE+Vest condition were encouraged to provide feedback on the cooling vest via an additional open-ended question, “What challenges have you encountered with wearing a cooling vest?”.

The experimental trial would be terminated if the participant met any of the following criteria: (i) requested to terminate the exercise session, (ii) completed the assigned physical activity, (iii) was unable to continue the assigned physical activity voluntarily, (iv) reported symptoms such as nausea, headache, dizziness, or cold clammy skin, or (v) showed signs of lethargy.

### Measurement, instrumentation, and calculation

During the trial, T_c_ was continuously recorded using the ingestible thermoregulatory pill. Four telemetric iButtons (Maxim Integrated, San Jose, CA, USA) were placed on the skin at the chest (T_chest_), bicep, thigh, and calf to calculate T_sk_ continuously (23). A chest-based HR monitor (Polar H10; Polar Electro Oy, Kempele, Finland) recorded HR. Ambient temperature and humidity were monitored using a heat stress monitor (QUESTemp°34; TSI Incorporated, Shoreview, MN, USA).

**Figure 2:**
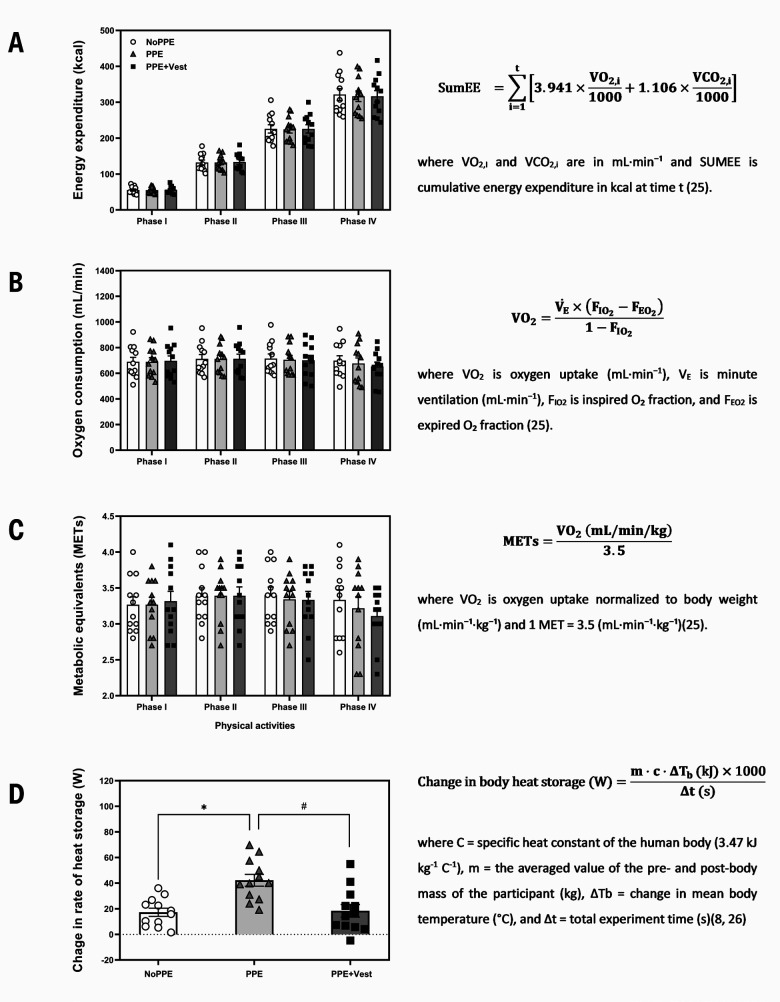
Metabolic response and equations. A. Energy expenditure, B. Oxygen consumption, C. Metabolic equivalents, and D. Change in body heat storage across the different physical activity phases in each of the conditions. The white circles, gray triangles, and black rectangles denote the individual data NoPPE, PPE, and PPE+Vest, respectively. Bar graphs represent the mean values with error bars indicating the SE for each condition.

Metabolic parameters, including energy expenditure (EE), oxygen utilization (VO_2_), and metabolic equivalent (MET), were continuously recorded using a Cardio-Pulmonary Exercise Testing machine (VyntusTM CPX Metabolic cart; Vyaire Medical, Mettawa, IL, USA) and a Pluto® treadmill (HP Cosmos, Traunstein, Germany). All equations utilized in this study are illustrated in figure 2. Perceptual outcomes included rating of perceived exertion (RPE) (24), thermal sensation (TS) (25), and thermal comfort (TC) (26) were recorded every 5 minutes during the assigned physical activities.

### Data analysis

The sample size for the study was determined using G*Power (Version 3.1, Heinrich-Heine-Universität Düsseldorf, Germany), with alpha and beta values set at 0.05 and 0.8, respectively. Based on previous studies assessing metabolic responses (η^2^ = 0.79) via two-way repeated-measures ANOVA and core temperature differences (η^2^ = 0.41) via t-test (11, 27), 12 participants were required, accounting for a 25% dropout. This also allows for gender comparisons (6 participants per group). Physiological and perceptual outcomes across conditions were analyzed with one-way ANOVA or two-way repeated-measures ANOVA for condition, time, and interaction effects. Ordinal data (RPE, TS, TC) were assessed using Friedman’s two-way analysis by ranks. Sex differences were analyzed with two-way ANOVA (sex × conditions) Statistical significance was set at P<0.05. Data are presented as mean (SD) (SPSS version 25, IBM, Armonk, NY, USA).

## Results

### Physiological and perceptual responses at baseline measurement

Baseline measurements were obtained ten minutes (T_c_ and T_sk_) and 5 minutes (HR, RPE, TS, and TC) before participants donned PPE and the cooling vest. All physiological and perceptual variables were comparable across the three conditions. T_c_ was consistent across trials [NoPPE: 37.5 (SD 0.3) °C, PPE: 37.5 (SD 0.2) °C, PPE+Vest: 37.5 (SD 0.1) °C], as was T_sk_ [NoPPE: 33.6 (SD 1.1) °C, PPE: 33.6 (SD 0.7) °C, PPE+Vest: 33.5 (SD 0.9) °C]. HR were also similar [NoPPE: 101 (SD 6) bpm, PPE: 98 (SD 9) bpm, PPE+Vest: 96 (SD 11) bpm), along with RPE (NoPPE: 0.1 (SD 0.3), PPE: 0.1 (SD 0.3), PPE+Vest: 0.1 (SD 0.3)], TS [NoPPE: 0.3 (SD 0.5), PPE: 0.3 (SD 0.5), PPE+Vest: 0.2 (SD 0.4)), and TC (NoPPE: 0.1 (SD 0.3), PPE: 0.3 (SD 0.5), PPE+Vest: 0.1 (SD 0.3)].

### Thermoregulatory outcomes

**Figure 3:**
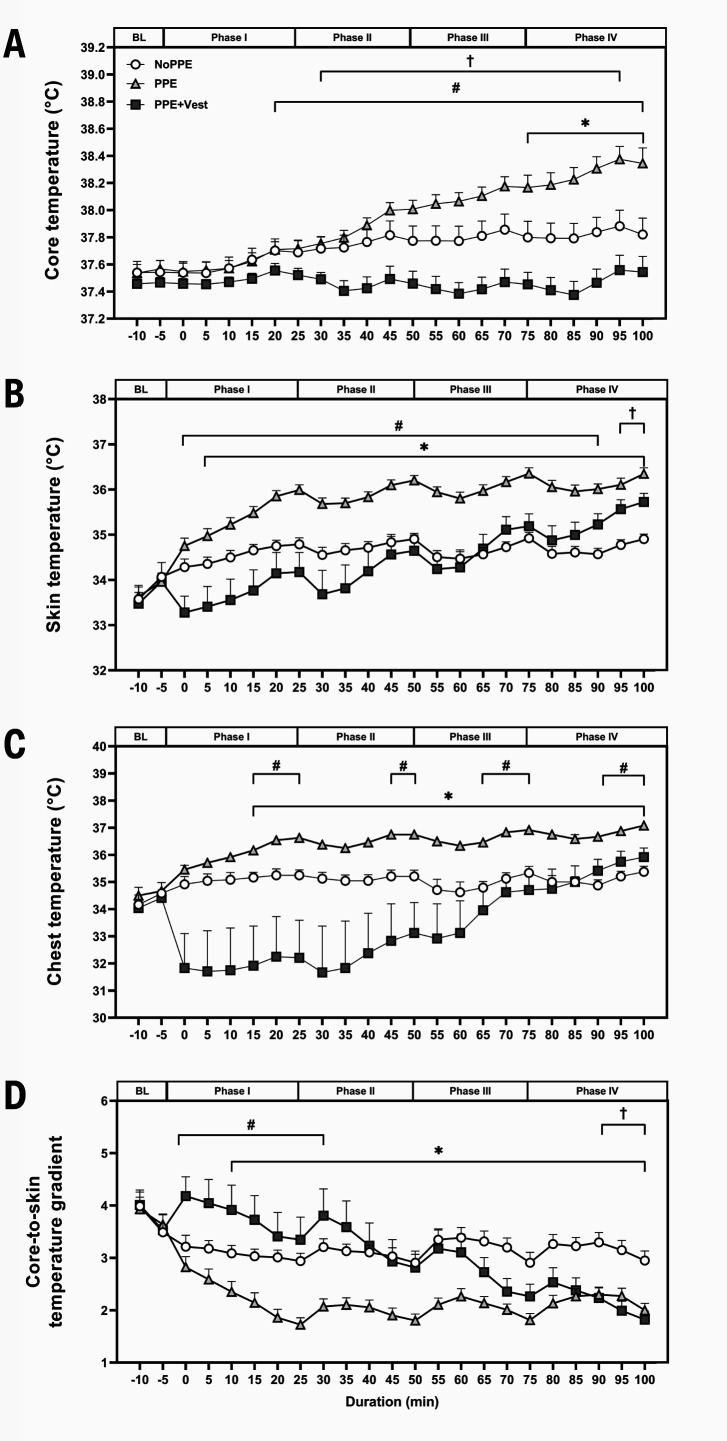
Thermoregulatory responses. A. Core temperature, B. Mean skin temperature, C. Chest temperature, and D. Core-to-skin temperature gradient. Baseline thermoregulatory measurements were conducted 10 minutes after the skin temperature data loggers were attached and before PPE and cooling vest donning. BL was shortened from baseline measurement. Each data point represents the mean with error bars indicating standard error (SE) for each condition. *P<0.05 between NoPPE and PPE, †P<0.05 between NoPPE and PPE+Vest, and ^#^P<0.05 between PPE and PPE+Vest.

*Core temperature.* T_c_ was higher in PPE during phase IV (P=0.011–0.046) compared to NoPPE. In contrast, T_c_ in PPE+Vest was lower than in PPE in phases I–IV (P<0.001–0.011) (figure 3A). Additionally, T_c_ in PPE+Vest was also lower than NoPPE in phases II–IV (P=0.001–0.049). Significant effects of condition, time, and interaction (P<0.001) were observed in the T_c_ response.

*Mean skin temperature.* A higher T_sk_ was observed in phases I–IV in PPE compared to NoPPE (P<0.001–0.029). A lower T_sk_ was also detected in phases I–IV in PPE+Vest compared to PPE (P=0.005–0.028). T_sk_ in PPE+Vest was comparable to NoPPE during phases I–IV. However, during the last five minutes of phase IV, the mean T_sk_ in PPE+Vest became higher than in NoPPE (P=0.015–0.022) (figure 3B). Additionally, the main effects of condition, time, and interaction (P<0.001) were observed in T_sk_ response.

*Chest temperature.* Higher skin T_chest_ was noted in all phases in PPE compared to NoPPE (P<0.001 to P=0.008). While T_chest_ was similar between NoPPE and PPE+Vest, intermittently higher T_chest_ was recorded in PPE than in PPE+Vest during all phases (P=0.015–0.046) (figure 3C). Additionally, significant main effects of condition (P=0.005), time (P<0.001), and interaction (P<0.001) were observed in T_chest_ responses.

*Core-to-skin temperature gradient.* A lower core-to-skin temperature gradient (T_gradient_) was detected in PPE than NoPPE in phases I–IV (P<0.001–0.033). Meanwhile, a higher PPE+Vest T_gradient_ in phases I–II (P=0.015–0.047) compared to PPE. However, there was a comparable T_gradient_ between NoPPE and PPE+Vest during phases I–III. After that, a lower T_gradient_ was observed in PPE+Vest than NoPPE during the last 10 minutes of phase IV (P<0.001) (figure 3D). In addition, the main effects of the condition (P=0.008), time (P<0.001), and interaction (P<0.001) were detected in T_gradient_ response.

### Sex difference in thermoregulation

**Figure 4:**
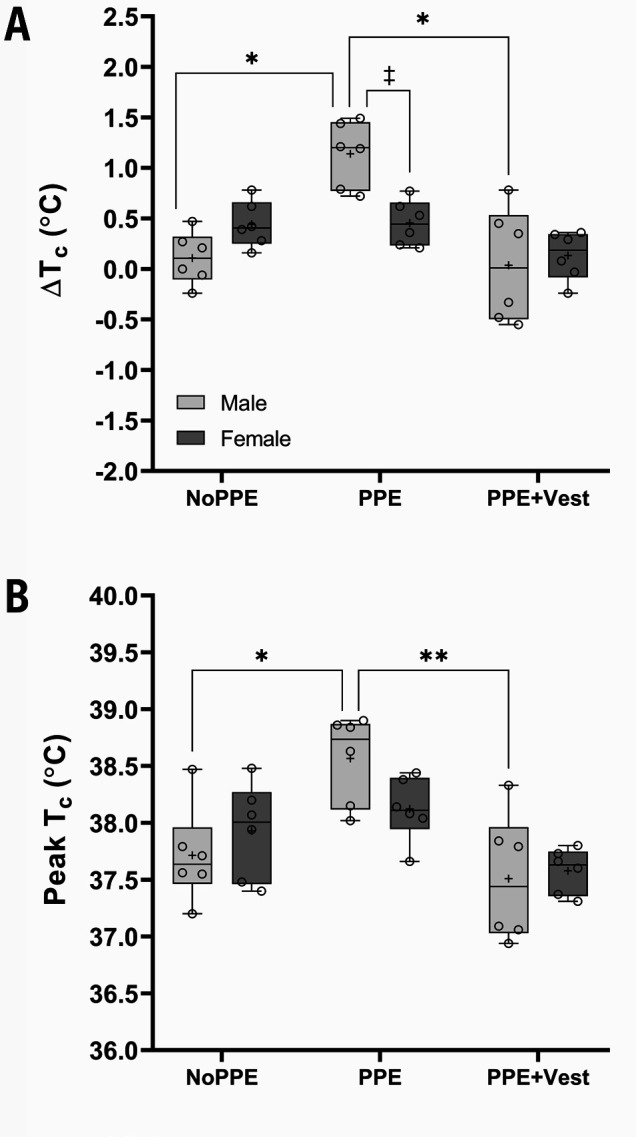
Sex difference. A. ΔT_c_ and B. Peak T_c_ during each condition, stratified by sex. Open circles represent individual data points. Boxes represent the interquartile range (25^th^ to 75^th^ percentiles), with the horizontal line indicating the median value. The + symbol represents the mean value for each dataset. Whiskers extend to the minimum and maximum observed values within each group. *P<0.05 between males, **P<0.001 between males, and ^‡^P<0.05 between both sexes.

A greater core temperature change (ΔT_c_) was observed among males than females during PPE [male: 1.1 (SD 0.3) °C versus female: 0.5 (SD 0.2 °C), P=0.016]. ΔT_c_ (NoPPE: 0.1 (SD 0.3) °C and PPE+Vest: 0.04 (SD 0.6) °C, P<0.001) (figure 4A) and peak T_c_ [PPE: 38.6 (SD 0.4) °C versus NoPPE: 37.7 (SD 0.4) °C and PPE+Vest: 37.5 (SD 0.6) °C, P=0.011 and P=0.001] (figure 4B) were higher during PPE than NoPPE and PPE+Vest in males. In contrast, comparable ΔT_c_ and peak T_c_ were observed across three conditions among females. The main effect of condition was highlighted for ΔT_c_ and peak T_c_ (P<0.001). An interaction effect was noted only in ΔT_c_ (P=0.002). No significant effect of sex was observed in ΔT_c_ and peak T_c_ responses.

### Metabolic outcomes

Consistent metabolic responses were observed across all three conditions (EE, VO_2_, and MET)(figure 2A-2C). The time effect was detected for EE (P<0.001), VO_2_ (P=0.022), and METs (P=0.036), however, there were no effects of condition and interaction for all parameters. Furthermore, lower change in body heat storage (figure 2D) was observed in NoPPE [17.3 (SD 11.0) W, P*=*0.001] and PPE+Vest [18.4 (SD 17.1) W, P=0.001], compared to PPE [42.3 (SD 15.9) W].

### Cardiovascular and perceptual outcomes

**Figure 5:**
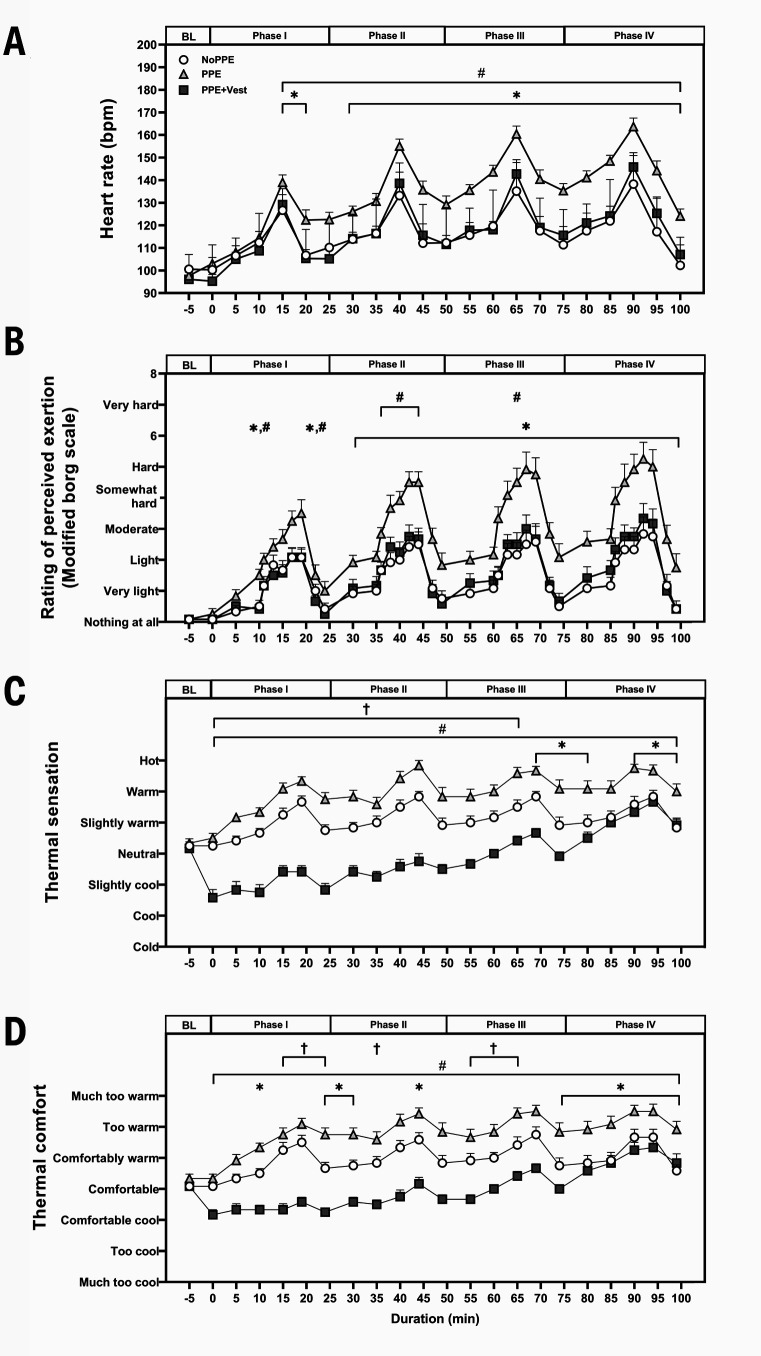
Heart rate and perceptions. A. Heart rate, B. Rating of perceived exertion, C. Thermal sensation, and D. Thermal comfort. Each data point represents the mean with error bars indicating SE for each condition. *P<0.05 between NoPPE and PPE, †P<0.05 between NoPPE and PPE+Vest, and ^#^P<0.05 between PPE and PPE+Vest

*Heart rate.* In the PPE trial, HR consistently showed higher values in phase I–IV compared to NoPPE (P<0.001–0.003) and PPE+Vest (P<0.001). In contrast, a similar HR was observed throughout all phases in NoPPE and PPE+Vest (figure 5A). The main effects of the condition, time, and interaction (P=0.001) were detected in HR during all phases.

*Rating of perceived exertion.* PPE demonstrated a higher RPE than NoPPE at phase I and phases II–IV (P=0.002–0.036). Additionally, the RPE in PPE was also greater than in PPE+Vest during walking in some minutes in phase I, phase II, and phase III (P=0.002–0.010). Furthermore, similar RPE levels were noted in NoPPE and PPE+Vest throughout all phases (figure 5B). Significant main effects of condition (P<0.001) were identified for the RPE. However, the time effect was only observed in the PPE and PPE+Vest conditions (P<0.001), but not in the NoPPE condition.

*Thermal sensation, thermal comfort, and subjective feedback.* Lower TS was observed during phases III and IV (P=0.010–0.030) in NoPPE than PPE. Intermittent lower TC was also detected in phases I, II, and IV (P=0.014–0.030) in NoPPE than PPE. Lower TS (P<0.001–0.023) and TC (P<0.001) were also detected in PPE+Vest than PPE during all phases, except for TC in phase IV. In addition, lower TS (P=0.008–0.011) and TC (P=0.008–0.011) were highlighted during all activities from phase I–III in PPE+Vest than NoPPE until no difference was detected during phase IV (figure 5C and 5D). The main effects of conditions of TS and TC were noted at P<0.001. The main effect of time was observed in PPE (TS: P=0.027 and TC: P=0.031) and PPE+Vest (TS and TC: P<0.001) conditions.

Of 12 participants, 8 (2 males and 6 females) reported that the cooling vest limited their trunk movements, such as flexion and rotation. Male and female participants also offered differing comments about the weight. Half of the females felt that the cooling vest was heavy, while the males did not.

## Discussion

This study evaluated the efficacy of carbon-based cooling vests in mitigating physiological and perceptual strain associated with heat and PPE use. The vest significantly reduced T_c_, T_sk_, HR, RPE, TS, and TC compared with PPE alone and produced responses comparable to the NoPPE condition, underscoring its capacity to maintain thermoregulatory stability without imposing additional metabolic demands.

Our results indicate that wearing PPE led to higher T_c_ and T_sk_, and lower T_gradient_. Participants’ T_c_ in PPE increased by 0.8 °C and reached 38.4 (SD 0.4) °C by the end of the activities. This T_c_ was comparable to a previous study, exceeding 38.0 °C (28). Prior research reported T_c_ elevations of 0.3 °C after 2-h PPE wearing (T_db_ of 30.2 °C) (10). Similarly, T_c_ increased by 0.6 °C in 1-h field settings at ambient temperatures of 36.6 °C (11) and by 0.4 °C during a 4-hour test at an ambient temperature of 28 °C (29), respectively. Work duration, intensity, and ambient temperature differences likely influenced T_c_ variation across studies. Greater heat strain is expected with prolonged exposure or higher workloads.

Despite the thermoregulatory strain, metabolic demands remained unchanged across conditions. The PPE load of 1.13 kg (≈1.6–2.3% of body mass) was likely too small to affect metabolism, consistent with evidence that loads <5% of body mass have minimal metabolic impact (30). Instead, PPE primarily impairs convective and evaporative cooling, reducing heat loss. In response, the body increases cutaneous vasodilation and skin blood flow to dissipate heat, which elevates Tsk and HR as circulation supports both thermoregulation and metabolism. Thus, the heightened heat strain with PPE was driven mainly by impaired heat dissipation rather than increased metabolic heat production.

Although the PPE+Vest added 4.13 kg (≈5.8–8.4% of body mass), exceeding the 5% threshold generally considered to affect metabolic responses, no increase in metabolic demand was observed. This paradox may be explained by the vest’s cooling capacity, which improved heat dissipation, reduced body heat storage, and offset the potential metabolic cost of the additional load (31). Importantly, the vest load was passively carried rather than actively manipulated, minimizing muscular effort and further limiting its metabolic impact. These results are consistent with previous studies reporting similar metabolic responses across control and cooling vest trials despite differences in vest weight (7, 12, 32). Collectively, our findings suggest that the thermal benefits of cooling vests outweigh their mechanical load, enabling healthcare workers to reduce heat strain without additional metabolic penalties, even when vest weight surpasses the 5% body mass benchmark.

Our findings show that the new carbon-based cooling vest effectively lowered T_sk_, T_chest_, T_c_, and HR compared to PPE alone. The reductions in T_sk_ (0.5–2°C) and T_chest_ (1.0–3.6 °C) were greater than those reported in previous studies using ice or PCM vests (0.8–1.5 °C and 1.0–1.2 °C, respectively), indicating improved local heat dissipation under the vest area (7, 8, 33). Notably, T_c_ remained stable throughout 100-min of PPE+Vest use, whereas earlier studies reported increases of 0.3–0.5 °C among healthcare workers wearing ice or liquid cooling vests with PPE (8, 33). These differences likely result from adding carbon additives to the PCM, which are known to improve thermal conductivity and stability by forming a conductive network within the material (16, 18). This property may have allowed faster and more even heat transfer from the skin to the vest. Reported thermal conductivity values for carbon-enhanced PCM (≈34.7 W·m^−1^·K^−1^) are much higher than those of water (≈2.35 W·m^−1^·K^−1^) (8, 16, 33), indicating a better ability for heat transfer and more effective T_sk_ reduction. Consequently, this skin cooling promotes greater heat dissipation from the body’s core to periphery. This mechanism helps maintain stable T_c_ during prolonged PPE use while performing activities. Although our study did not directly measure heat flux or vest–skin temperature gradients, the observed reductions in T_sk_, T_gradient_, T_c_, and ΔT_b_ provide indirect evidence that the carbon vest improved heat dissipation. Such physiological responses serve as an indicator of enhanced heat transfer ability. However, we acknowledge that the exact mechanisms are still theoretical. The increased cooling effect is probably due to both the material properties and the structural design of the vest. Future research involving direct heat flux measurements and comparisons with conventional PCM or water-based vests is necessary to verify the physical basis of these benefit.

In this study, males exhibited greater heat strain when wearing PPE, as indicated by a larger ΔTc compared with females. A higher LBM in males likely contributed to greater metabolic activity and excess heat accumulation (34). As a result, men began with a higher heat burden, and the absolute reduction in Tc achieved with the cooling vest was larger among males than females (1.1 °C compared with PPE). In contrast, females maintained relatively stable Tc across NoPPE and PPE conditions. Although women generally started with a slightly higher Tc, potentially influenced by hormonal status (35), their lower LBM and higher BSA/LBM ratio likely facilitated more efficient heat dissipation relative to heat production. Consequently, Tc did not increase markedly under PPE, and the absolute reduction observed with the cooling vest appeared smaller among females.

During PPE+Vest, greater reductions in T_sk_ and T_chest_, contributed to lowering HR, which aligns with previous studies showing that cooling decreases circulatory strain during work in the heat (8, 32, 33). By lowering skin temperature, the vest likely increased the thermal gradient and improved heat transfer. Previous studies have shown that reductions in skin temperature result in lower cutaneous blood flow requirements, thereby easing circulatory strain (36, 37). Importantly, this physiological relief translated into perceptual benefits. These findings align with prior research showing that PPE worsens perceptual strain (38, 39), while cooling interventions mitigate these effects (9, 33). Cooling vests shifted TS from “warm/hot” toward “neutral or slightly cool” and improved TC, maintaining participants in “comfortable” to “comfortably cool” throughout prolonged exercise. Such perceptual improvements are critical in occupational settings, as they may sustain motivation, concentration, and task performance under thermal stress.

Nevertheless, subjective feedback also highlighted drawbacks of the current vest design. Several participants, particularly females, noted restrictions in movement and heavier sensations, which may be linked to anatomical differences and the relative coverage of cooling packs. Solid and multiple PCM packs can reduce flexibility, limit the range of motion, and potentially affect task performance. These limitations suggest that future vest designs should prioritize not only thermal efficiency but also ergonomics, comfort, and usability across diverse user groups.

### Limitations

This study has several limitations. First, we did not directly compare the carbon-based cooling vest with other conventional cooling vests, nor did we quantify the vest’s surface temperature, skin-contact temperature, or cooling duration. These parameters are important for understanding the thermodynamic characteristics and heat transfer efficiency of the vest. Additionally, heat flux and physiological heat exchange mechanisms were not measured. Future studies should therefore include direct physical and physiological comparisons with traditional cooling vests and incorporate mechanistic measurements to validate and extend the present findings. Second, testing was conducted in a controlled laboratory environment, which may not reflect real-world conditions where factors such as wind, sunlight, humidity, and variable activity levels influence heat strain. Field studies are therefore warranted, particularly among healthcare workers operating in hot environments. Third, female participants were tested outside of menstruation without objective confirmation of hormonal phase. Small cycle-related variations may have influenced responses. Finally, the relatively small sample size limited the ability to detect sex differences and reduced generalizability. Larger studies are needed to confirm and extend these findings.

### Concluding remarks

Our findings provide practical insight into managing heat strain when using PPE. Wearing PPE in hot conditions markedly increased physiological and perceptual strain. However, the novel carbon-based cooling vest effectively mitigated physiological and perceptual strain for up to 100 minutes, which closely resembled the NoPPE condition. The additional weight of the PPE and vest did not elevate metabolic demands, underscoring the feasibility of integrating cooling strategies without imposing extra physiological cost.

## Data Availability

The data used in this study are confidential and not publicly available due to data protection regulations. Requests for data access may be directed to the corresponding author, subject to approval by the relevant ethics committees and data custodians.
